# Rare Sighting of Posterior Communicating Artery Infundibulum With Posterior Cerebral Artery Origin

**DOI:** 10.7759/cureus.46322

**Published:** 2023-10-01

**Authors:** Naveen K Suryadevara, Dan Y Draytsel, Neil C Suryadevara, Adam R Blanden, Hesham Masoud

**Affiliations:** 1 Neurology, Upstate University Hospital, Syracuse, USA

**Keywords:** ct angio, variant, neurovascular, anatomy, cerebrovascular

## Abstract

Infundibula are funnel-shaped lesions that develop at the intersections of major intracranial arteries. These lesions are prone to being misdiagnosed as intracranial aneurysms. The most common arterial infundibula have been noted in the posterior communicating artery (PCoA) branch of the internal carotid artery (ICA). Digitally subtracted angiography performed included catheter angiography of the vertebral artery and ipsilateral carotid to evaluate the suspected lesion. Right vertebral angiography demonstrated an infundibulum seen at the right PCoA/posterior cerebral artery (PCA) junction, with noted posterior-to-anterior circulation dominance of the Circle of Willis collateral flow. We report a case of posterior communicating artery infundibulum arising from the posterior cerebral artery origin in a 38-year-old man.

## Introduction

The posterior communicating artery (PCoA) branch of the internal carotid artery (ICA) is the most common site of cerebral infundibula [1]. Infundibula are similar in appearance to aneurysms, leading to frequent misdiagnoses. Morphologically, aneurysms take a variety of appearances resulting from the abnormal arterial dilation of a blood vessel. These abnormalities result in turbulent flow, weakening of the vessel, and rupture. By contrast, infundibula assume a funnel shape at the origin of a vessel, preserve laminar flow, and are commonly seen on otherwise unremarkable angiograms [2]. There have been rare case reports of infundibula rupture, prompting some to describe them as pre-clinical lesions; however, this remains controversial and is significantly less common than true aneurysm rupture [2]. PCoA from the ICA origin appears to have a higher degree of susceptibility to the presence of infundibula when compared with other cerebral arteries, with 67.4% of infundibula occurring there [3]. Thus far, there has been no literature describing infundibula occurring at the posterior cerebral artery (PCA) origin of the PCoA. In this report, we present a patient with an infundibulum on the right PCoA from the right PCA masquerading as a mycotic aneurysm in a patient with bacterial endocarditis.

## Case presentation

**Figure 1:**
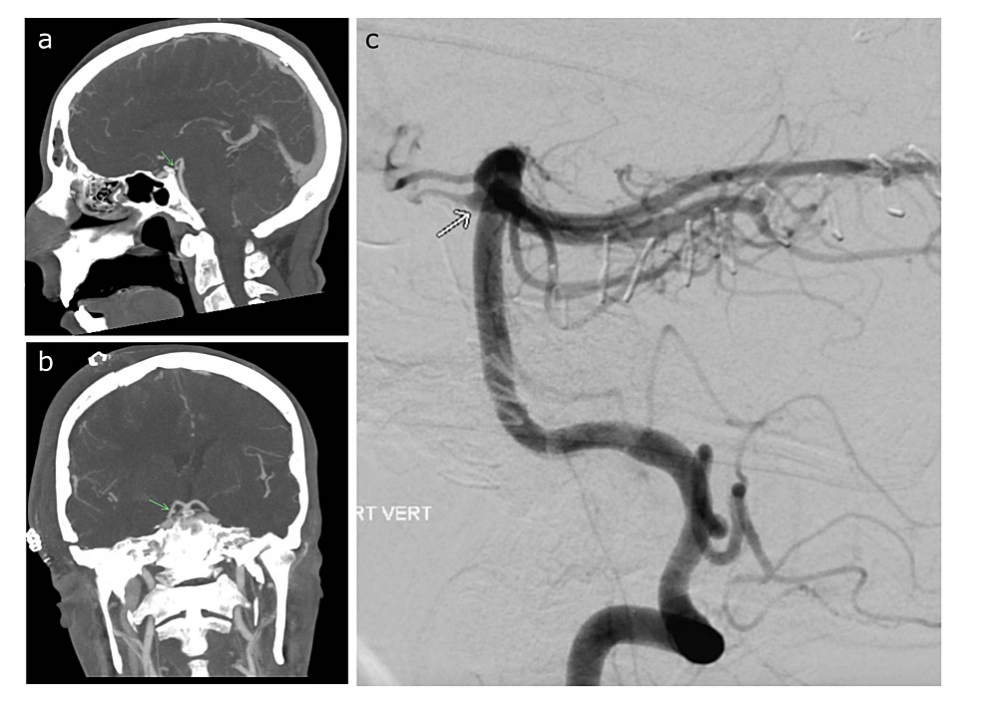
CT angiogram of the head 8.0 mm maximum intensity projections in (a) the sagittal and (b) the coronal planes demonstrating a dilation concerning for aneurysm arising from the right PCA (arrow); (c) DSA of the right vertebral artery in lateral projection demonstrating posterior communicating artery infundibulum arising from the proximal (P1-segment) posterior cerebral artery (arrow) PCA: posterior cerebral artery; DSA: digital subtraction angiography

A 38-year-old man with a noted history of intravenous drug use presented with acute right-sided headache, fever of 39.2, leukocytosis (WBC 22.6 x103, ANC 20.1) and encephalopathy, Glasgow coma scale of 10 (Eyes 2, Verbal 2, Motor 6). CT head was notable for bilateral subdural collections. Lumbar puncture revealed an infectious profile (TNC: 400 cells/uL, 88% neutrophils, 7% monocyte/macrophage; RBC: 52 cells/uL, glucose 51 mg/dL, protein 120 mg/dL). The patient was brought to the operating room for subdural evacuation. Blood, CSF, and surgical cultures grew methicillin-resistant Staphylococcus aureus (MRSA), confirming the diagnosis of subdural empyema due to MRSA, and the patient was started on pulse intravenous vancomycin. The patient’s hospital course was complicated by embolic-appearing infarcts on serial MRIs, multifocal lung nodules with central necrosis and air-fluid levels on CT thorax with contrast consistent with septic emboli, and persistently positive blood cultures despite treatment with vancomycin. After a thorough but unrevealing search for a structural embolic source including extremity dopplers, transthoracic, and transesophageal echocardiography, the patient was diagnosed clinically with bacterial endocarditis. The patient was recovering well in the inpatient neurology unit when a cerebral angiogram was requested for evaluation of a possible proximal PCA aneurysm at the P1/P2 junction, extending posteriorly, seen on surveillance CT angiography (Figure 1). Given the clinical context of bacterial endocarditis and the evidence of systemic emboli, concern for a mycotic aneurysm was raised. Indeed, up to 5-10% of patients with bacterial endocarditis will develop mycotic aneurysm, and MRSA is among the most common culprit pathogens [4].

Digital subtraction angiography (DSA) performed included catheter angiography of the vertebral artery and ipsilateral carotid to evaluate the suspected lesion. Right vertebral angiography demonstrated an infundibulum at the right PCoA/PCA junction (Figure 1), with noted posterior-to-anterior circulation dominance of the Circle of Willis collateral flow (Figure 1, Video 1). There was no evidence of contrast stasis or other aneurysmal dilation concerning mycotic aneurysm. The patient completed a course of intravenous antibiotics while in the inpatient service and was discharged to a rehabilitation facility without further complications. His outpatient course was complicated by the development of symptomatic localization-related epilepsy now treated with valproic acid. MRI of the brain with and without contrast at 1 year demonstrated no evidence of new infarct or hemorrhage. At the 14-month follow-up, there were no neurovascular events or complications noted.

**Video 1:** Right vertebral angiography with infundibulum at the right PCoA/PCA junction PCoA: posterior communicating artery; PCA: posterior cerebral artery

## Discussion

Infundibula are commonly encountered anatomical variants present in the cerebral arterial circulation and represent limited pathological consequences [1]. Infundibula arising from the ICA origin of the PCoA are common; however, those arising from the PCA origin are exceedingly rare, and at the time of writing we are unaware of any previous report of this variant [5]. The cause of its rarity is unclear and is likely reflective of individual anatomic variance of the Circle of Willis. The posterior to anterior flow dominance demonstrated in this patient offers an explanation that underlies the PCA origin of this PCoA infundibulum (Video 2).

**Video 2:** Posterior to anterior flow dominance offering an explanation that underlies the PCA origin of this PCoA infundibulum PCoA: posterior communicating artery; PCA: posterior cerebral artery

This is distinct from the common infundibula seen in the communicating ICA segment, as most patients demonstrate usual anterior to posterior dominance of flow [3]. The only other case we have encountered of an infundibulum arising from the PCA in otherwise normal posterior circulatory anatomy demonstrated an infundibulum arising from the proximal P1 segment of the PCA at the origin of two thalamoperforators mimicking an aneurysm [6]. Although not involving the PCoA, this lesion would presumably share the flow dominance originating from the P1 into downstream arteries in which the infundibulum developed, as in this case. With the risk of mycotic aneurysm in this case it was critical to differentiate infundibulum from pathology, and in the absence of prior vessel imaging for comparison, the patient underwent a catheter study and identification of the defect, as an infundibulum saved the patient from unnecessary PCoA sacrifice with potentially disastrous clinical consequences from branch occlusion. The patient was discharged with outpatient follow-up and experienced no other sequelae. PCoA infundibula are infrequently malignant, particularly those sized smaller than 3 mm, with the chance of aneurysmal progression unlikely when follow-up vessel imaging is completed in appropriate intervals with demonstrated stability [7].

## Conclusions

We report a case of a PCoA infundibulum arising from the posterior cerebral artery origin. Although rare, multiple reports now exist of infundibula arising in normal posterior circulation anatomy in addition to their common locations in the anterior circulation, raising the possibility that they may arise at any vessel origin. Interventionalists need to remain vigilant of this possibility when assessing such lesions to avoid the unnecessary risk of overtreatment.
